# Effect of Tai Chi Compared to Running on Drug Cravings, Attention Bias, and Physical Fitness in Men with Methamphetamine Use Disorder

**DOI:** 10.3390/healthcare12161653

**Published:** 2024-08-20

**Authors:** Ling Zhang, Haiqi Zeng, Yuliang Sun, Huiheng Xue, Liquan Gao, Wenfei Zhu

**Affiliations:** School of Physical Education, Shaanxi Normal University, Xi’an 710119, China; zhangling1@snnu.edu.cn (L.Z.); zenghq@snnu.edu.cn (H.Z.); ysun@snnu.edu.cn (Y.S.); xuehuiheng@snnu.edu.cn (H.X.)

**Keywords:** methamphetamine use disorder, Tai Chi, attention bias, physical fitness

## Abstract

Background: Methamphetamine use disorder (MUD) is a global health problem. Studies have shown Tai Chi is a potential treatment for MUD. We aimed to explore the effectiveness of Tai Chi in improving drug cravings, attention bias, and physical fitness in men with MUD compared with aerobic exercise. Methods: A total of forty-eight participants (mean age 39.1 ± 8.7 years) were randomly assigned to either the Tai Chi group (TC) or the running group (RG). The TC performed 60 min of moderate-intensity (65–75% HR_max_) Tai Chi exercise three times a week. The RG performed 60 min of moderate-intensity (65–75% HR_max_) running on a treadmill three times a week. Before and after the intervention, drug cravings, attention bias, and physical fitness were evaluated. Results: After 12 weeks, we found the TC significantly improved in attention bias (F (1, 43) = 6.023, *p* = 0.019, d = −0.42) and reaction time (F (1, 43) = 6.181, *p* = 0.017, d = −0.72). No significant improvement was found in other variables in the TC, compared to the RG (*p* > 0.05). Conclusions: The 12-week Tai Chi intervention improved attention bias and reaction time, compared to RG. Tai Chi exercise might be a potential auxiliary method for the rehabilitation for men with MUD.

## 1. Introduction

Substance use disorder (SUD) is a chronic disease characterized by drug seeking and use that is compulsive or difficult to control despite harmful consequences. Methamphetamine is one of the most commonly abused substances. The use of methamphetamine is still on the rise, posing significant risks to both physical and mental health [1]. The World Drug Report 2022 shows that about 36 million people worldwide are addicted to drugs, but only one in seven can get treatment [2]. Prolonged drug abuse will seriously damage physical and mental health [3], causing diseases of the central nervous system, cardiovascular system, and other health issues [4].

Long-term methamphetamine use can cause cognitive brain damage, particularly in inhibitory control, working memory, and attention [5], which persists after detoxification [6]. Individuals with methamphetamine use disorder (MUD) often develop selective attention to cues related to drug stimuli which is commonly referred to as attention bias. The cognitive processing model of addiction proposes that individuals with MUD have an attention bias towards stimuli related to drugs, which can lead to relapse [7]. Research has shown that MUD patients demonstrate significant attention bias towards drug-related cues, potentially due to dysfunction in the prefrontal cortex and amygdala, areas critical for decision-making and emotional regulation [8]. These cognitive impairments, including deficits in inhibitory control and working memory, not only persist after detoxification, but also significantly increase the risk of relapse into addiction [9]. Therefore, it is significant to promote physical and mental health in individuals with MUD [10].

Exercise training, which has economic, green, and side-effect-free features, is considered to be an effective means to promote the physical and mental rehabilitation of individuals with MUD [11,12]. Compared to traditional physical exercise, the mind–body exercise of Tai Chi has become increasingly popular worldwide. Rooted in Chinese medicine and originating in martial arts, Tai Chi has been developed for thousands of years. Tai Chi is characterized by gentle, flowing movements that emphasize the coordination of breathing with physical activity, embodying a balance of internal and external cultivation. The 24-form Tai Chi, one of the most common forms, consists of 24 continuous movements, each flowing into the next, designed to stimulate the body’s meridians and promote the flow of qi and blood. The practice not only increases physical flexibility and strength, but also balances emotions and improves mental health through sustained meditation and focused attention [13,14]. Studies indicate Tai Chi has been shown to have a more positive effect on cognitive function and mood than more traditional forms of aerobic exercise, such as running and swimming [15]. A meta-analysis found that adults who participated in Tai Chi had improved cognitive abilities compared to the control group [16]. Furthermore, Tai Chi has a positive impact on improving cognitive function [17], as well as promoting physical and mental recovery in individuals with MUD [18]. At the core of Tai Chi training are the 24 forms, which cover different parts of the body and improve muscle strength, joint flexibility, and balance through slow and controlled movements. Each movement focuses on natural body movement and coordinated breathing, with participants focusing on the precision of each movement and the rhythm of their breathing. The entire training process emphasizes “mind over body”, where inner intention guides the body’s movement, which can help improve attentional bias and reduce sensitivity to drug-related cues [19,20]. Additionally, the meditation component of Tai Chi, which is good for improving concentration, also involves learning choreographed patterns of movement that can enhance visual–spatial processing skills [16]. Based on these factors, Tai Chi has the potential to improve attention bias and reduce sensitivity to drug-related cues in individuals with MUD.

Aerobic exercise, such as running, has been shown to improve physical health, including cardiovascular fitness and body composition [21]. Running is also effective in reducing stress and anxiety, which are often elevated in people with MUD [22]. In terms of cognitive effects, research suggests that aerobic exercise, including running, can improve cognitive functions such as executive control, working memory, and attention [23]. For example, studies have shown that regular aerobic exercise can lead to improvements in reaction time, processing speed and overall cognitive flexibility [24]. However, the specific effects of running on attention bias and inhibitory control, which are critical for relapse prevention in MUD patients, are less well understood. Some studies suggest that while running may improve overall cognitive resilience, its effects on specific cognitive domains relevant to addiction recovery are not as pronounced as those observed with mind–body exercises such as Tai Chi [25]. In contrast, with its holistic approach that combines physical, cognitive, and meditative elements, Tai Chi may offer unique cognitive rehabilitation benefits [16]. This approach is particularly beneficial in addressing specific cognitive impairments associated with MUD, potentially reducing the likelihood of relapse.

Despite the promising aspects of Tai Chi, there is limited research comparing its effects with traditional aerobic exercises, such as running, on men with MUD. This study aims to explore the effectiveness of Tai Chi in reducing drug cravings, improving attention bias, and enhancing physical fitness in this population. Running was chosen as the control exercise due to its well-established cardiovascular and stress-reduction benefits, as well as its widespread accessibility and frequent use in therapeutic settings [26]. This choice provides a common baseline for comparing the specific benefits of Tai Chi. We hypothesize that the Tai Chi mind–body intervention will be more effective than running in reducing drug cravings, improving attention bias, and promoting physical fitness in men with MUD.

## 2. Materials and Methods

### 2.1. Participants

Forty-eight individuals (all men) were recruited to participate in this trial from the Drug Rehabilitation Center in Shaanxi Province, China. Participants fulfilling the following inclusion criteria were included in the study: (1) with methamphetamine use disorder diagnostic criteria according to the Diagnostic and Statistical Manual of Mental Disorders (DSM) IV; (2) currently incarcerated and remaining incarcerated until the end of the experiment; (3) having a normal physical condition as assessed by the Physical Activity Adaptability Questionnaire (PAR-Q) and being able to participate in moderate-intensity aerobic exercise; (4) normal cognitive status in the Mental Status Examination Scale (MMSE) screening; (5) participants who were under standardized management in a mandatory isolation detoxification center, which included a controlled and uniform diet to minimize the impact on cholesterol and blood pressure levels; (6) no recent use of medicines known to affect cholesterol or blood pressure. Participants were randomly divided into two groups, namely the Tai Chi group (TC, n = 24) or the running group (RG, n = 24). The sample size for this study was calculated using G-Power software (version 3.1.9.7, Heinrich Heine University, Düsseldorf, Germany). Prior to the experiment, the mean effect size was determined to be 0.45 in combination with previous studies [11], and the total sample size was calculated by G-Power to be 42 when setting a significance level of α = 0.05 and the power to be 0.80. Considering participant attrition (15%), the sample size was increased to 48 people. Demographic characteristics of the two groups are shown in Table 1 and there are no significant differences in the relevant indicators (*p* > 0.05). All participants were aware of the trial tasks and signed informed consent. The study was approved by the Scientific Ethics Committee at the Shaanxi Normal University (202316011).

### 2.2. Study Design and Procedure

**Figure 1:**
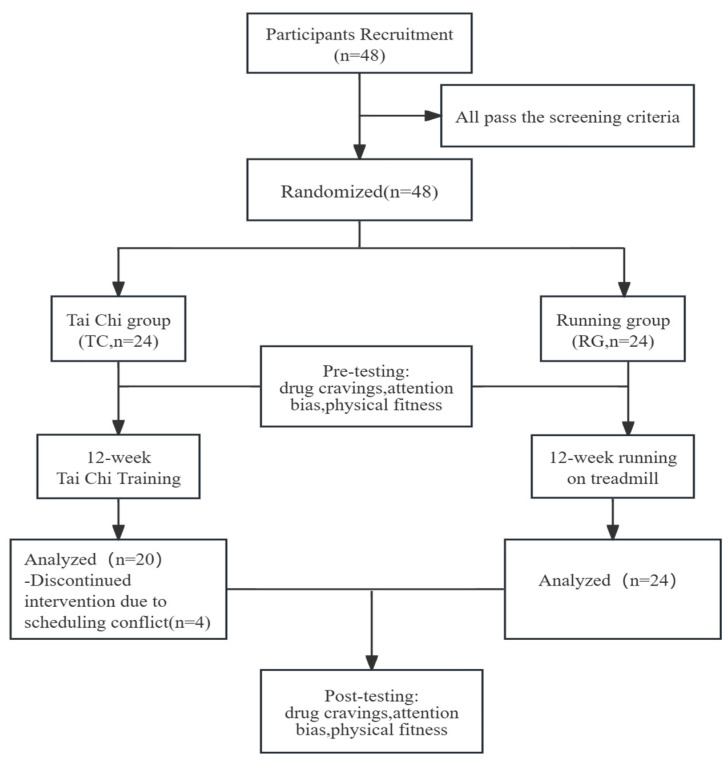
Overview of the study design.

The 48 participants included in the experiment were asked to complete a baseline test. The baseline measurements included drug cravings, attention bias, and physical fitness. During the intervention, the TC conducted moderate-intensity Tai Chi training that lasted for 12 weeks (exercise lasted 60 min three times a week). The running group underwent 12 weeks (exercise lasted 60 min three times a week) of moderate-intensity running on a treadmill. To track changes, the identical post-test was administered after 12 weeks. Four participants in the TC were unable to participate the post-test due to scheduling conflicts with other rehabilitation treatments, resulting in a final sample size of 44 individuals included in the data analysis (Figure 1).

### 2.3. Intervention

Each training session consisted of 60 min of Tai Chi or running intervention that began with 5 min of warm-up, followed by the main part (50 min) with moderate-intensity 24-form Tai Chi exercise or running on a treadmill, and 10 min for dynamic and static stretching after the Tai Chi or running training. The intervention lasted 12 weeks (exercise lasted 60 min three times a week). The Tai Chi sessions focused on performing the 24 forms, emphasizing coordination of movement and breathing, and maintaining a moderate intensity of exercise. These sessions were designed not only to improve physical aspects such as strength, flexibility, and balance, but also to enhance mental focus and reduce attentional bias by integrating mindful breathing and movement coordination. During the training, all participants wore heart rate monitors (Fit-Mao HW807, Shandong Delay Cook Health Care Equipment Co., Dezhou, China) to track exercise intensity in real-time to ensure the safety of the participants as well as a moderate level of exercise. Moderate intensity was defined as 65% to 75% HR_max_ based on previous research results, where HR_max_ was calculated by the formula HR_max_ = 206.9 − 0.67 × age [27].

### 2.4. Measures

#### 2.4.1. Drug Cravings

**Figure 2:**
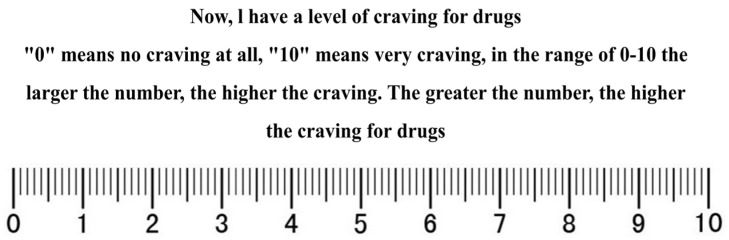
Visual analog scale for drug cravings.

The drug cravings of individuals with MUD were assessed using the visual analog scale (VAS) (Figure 2). The VAS, which is a Likert self-rating scale that uses a 10 cm line segment to indicate drug cravings with 11 score points, begins with a “0” for “not at all” and ends with a “10” for “extreme cravings”. During the test, the participants were given a simulated drug model and were asked to stare at it for 1–2 min, after which the drug cravings level was assessed by VAS. In the range of 0–10, as the number gets larger, the higher the participant’s cravings for drugs. This method has been widely recognized for its effectiveness in reflecting the subjective cravings of people with MUD and has been shown to have good validity and reliability, with a Cronbach’s alpha value typically reported to be around 0.86 to 0.89 [28,29,30].

#### 2.4.2. Attention Bias

**Figure 3:**
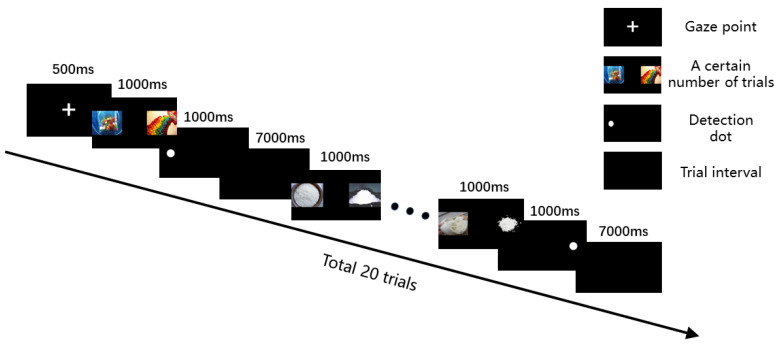
Flowchart of the visual dot-probe paradigm.

Individuals with MUD tend to pay more attention to drug-related stimuli than other stimulants, which is known as attention bias [7]. A series of neuropsychological tests allow for a quantitative assessment of an individual’s overall cognitive functioning [31]. Therefore, a visual dot-probe experimental paradigm based on E-prime 3.0 software (Psychology Software Tools, Sharpsburg, PA, USA) was designed in this study to test the attention bias of individuals with MUD (Figure 3).

The visual dot-probe paradigm was a measure of the participant’s attention biases [32]. The pictures required for the experiment were obtained from the Chinese Affective Picture System (CAPS), and 40 pictures (20 neutral pictures and 20 drug pictures) were identified after selection. All of the pictures were rated by 100 non-experimental participants on a nine-point scale in terms of validity and arousal [33]. The validity and arousal of the drug-stimulated pictures were 4.38 and 4.21, respectively, and those of the neutral-stimulated pictures were 5.05 and 3.57, respectively. The paradigm required the simultaneous presentation of a drug stimulus and a neutral stimulus, which appeared randomly 8 cm apart on the left and right sides of the screen. When both stimuli disappeared, a detection dot that lasted 1000 ms was presented randomly at both stimulus points. Participants were required to judge the location of the detection dot by pressing the “F” if it was on the left, otherwise pressing “J”. If participants responded slower when the probe dots appeared at the location of the drug stimulus compared to the area of the neutral stimulus, then it suggested an attention bias to the drug stimulus. After the experiment started, a gaze point “+” for 500 ms was presented in the center of the screen. The paradigm has 20 trials, each with an interval of 7000 ms. The attention bias score is an indirect indicator of participants’ drug cravings, which is determined by the response time difference between the congruent and non-congruent tests.

#### 2.4.3. Physical Fitness Test

In our study, the physical fitness status of men with MUD was measured using a variety of indicators. These indicators were chosen for their relevance to overall physical health and fitness, their practicality in a controlled environment, and their established validity and reliability in previous research [34]. The indicators included height, weight, body mass index (BMI), waist circumference, grip strength, lung capacity, standing on one foot with eyes closed, sitting forward bend, choice reaction time, and blood pressure and biochemistry measures.

The height and weight test used an intelligent physical examination all-in-one machine (iDong@TA106, Shenzhen Taishan Sports Technology Co., Ltd., Nanning, China). Participants were asked to stand barefoot in a natural standing position when conducting the test. The test results were automatically broadcasted by the machine, and we finally recorded the results in a table.

Each participant’s BMI (kg/m^2^) was calculated by their body weight (kg) divided by their height (m) squared.

Waist circumference was measured with a soft ruler, which was non-elastic and had a minimum scale of 1 mm. Participants were asked to stand naturally with their feet 25–30 cm apart and to breathe smoothly. The position to be measured was at the midpoint of the line connecting the highest point of the iliac crest and the lower edge of the 12th rib. This measurement is critical in assessing central obesity and related health risks [35].

In the lung capacity test, the electronic spirometer was used for measurement. Participants were asked to stand naturally, breathe deeply, and then exhale slowly until the gas was exhaled, without pausing during the blowing process. The test was performed twice continuously, and the maximum value was recorded. This is an assessment of the health and capacity of the respiratory system [36].

To measure grip strength, we used a handgrip dynamometer. The test includes the dominant hand twice clutching the instrument to the maximum force (no secondary force). The highest value was employed for the determination of the maximal grip strength. This measurement is a reliable indicator of overall muscle strength [37].

One-leg standing with eyes closed was measured with the traditional stopwatch timing method. The participants were asked to stand naturally on the mat barefoot. When they heard the “start” command and the timing started, they closed their eyes and stood on one leg with the dominant leg, while the non-dominant leg was lifted off the ground with could not touch the supporting leg. The test was usually measured twice in a row, and the best result was used for recording. This test assesses balance and proprioception [38].

Sit and reach was tested with a sitting forward bend tester. The participants were required to front the instrument with their body bent forward and their legs straight, and to push the cursor forward at an even speed with the fingers of both hands until they could not push any further. The test was performed twice, and the maximum value was recorded. This test is a common and validated method of assessing flexibility [39].

Choice reaction time was measured using an electronic reaction time tester (Nantong Yuejian Body Measurement Equipment Co., Ltd., Nantong, China). When the test started, a light dot appeared on the instrument, and the participants had to press the corresponding button. The instrument would finally show the average reaction time. The test was conducted twice a row, and the best result was recorded. This test measures cognitive and motor reaction times [40].

#### 2.4.4. Blood Pressure and Biochemistry Measurements

Blood pressure was measured using an electronic sphygmomanometer (OMRON Co., Ltd., Dalian, China) for diastolic and systolic blood pressure. The participants were seated quietly, and the cuff of the blood pressure monitor was wrapped tightly around the upper arm, with the lower edge 2–3 cm above the elbow. Blood pressure was measured three times and the average was recorded.

After participants had been fasting for 8 to 12 h, venous blood samples were collected in the hospital early in the following morning. An Automatic Biochemical Analyzer (BS-860, Meizu Medical International Co., Ltd., Zhuhai, China) performed a 10 min analysis of the blood sample at 3000 rpm, resulting in the final separation of the serum. Insulin, glucose, total cholesterol, triglycerides, high-density cholesterol (HDL-C), and low-density cholesterol (LDL-C) were measured in a regular medical laboratory.

### 2.5. Statistical Analysis

In this study, the independent variable was the type of intervention (Tai Chi or aerobic exercise), the dependent variables were drug cravings, attention bias, and physical fitness indicators, and the control variables were age, years of education, and baseline values of the dependent variables.

All collected data were processed by a software (SPSS Version 25.0, IBM Corporation, Armonk, NY, USA). The mean (M) and standard deviation (SD) of all variables were calculated. The normal distribution of baseline characteristics of participants was verified by the Shapiro–Wilk test. For quantitative outcomes, normality and homogeneity of variance were tested (*p* > 0.05). Baseline comparisons among groups were performed using independent t-tests or Chi-square tests.

Analysis of covariance (ANCOVA) was used to determine whether there was a difference between the intervention and control groups after 12 weeks of intervention, controlling for baseline values, age, and years of education. A *p*-value of <0.05 was considered to indicate a statistically significant difference. Additionally, Cohen’s d was used to further compare the differences between groups from baseline to post-test [41]. The magnitude of effect sizes was categorized as none (0 ≤ d < 0.20), small (0.20 ≤ d < 0.50), medium (0.50 ≤ d < 0.80), or large (d ≥ 0.80).

## 3. Results

### 3.1. The Effect of Tai Chi Exercise on Cravings of Men with MUD

**Figure 4:**
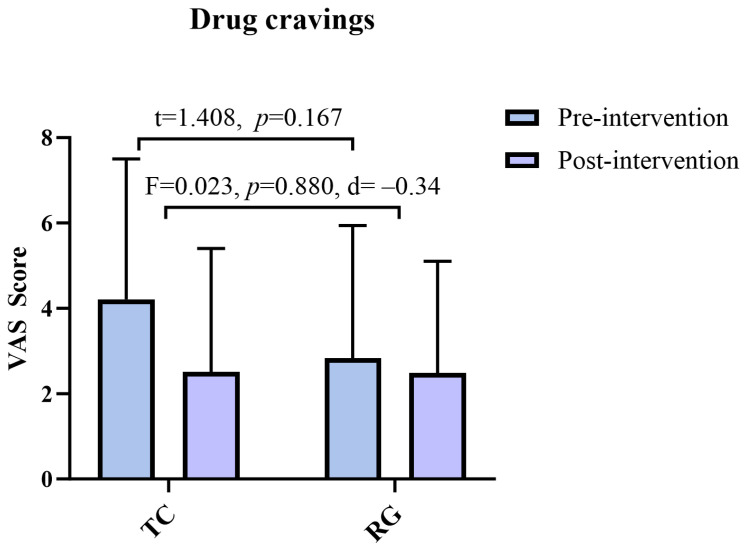
Comparison of drug cravings results between the TC and running group. Note: VAS: visual analog scale; TC: Tai Chi group; RG: running group.

There was no significant difference in baseline drug craving scores between the two groups (t = 1.408, *p* = 0.167). After 12 weeks of Tai Chi exercise intervention, no statistically significant difference in drug craving was found in the TC with a smaller effect size compared to the running group (F (1, 43) = 0.023, *p* = 0.880, d = −0.34) (Figure 4).

### 3.2. Influence of Tai Chi Exercise on the Attention Bias of Men with MUD

Table 2 shows the results of measuring attention bias in men with MUD using the visual dot-probe paradigm. No significant baseline difference was found in attention bias score and reaction time of neutral or drug pictures between groups at baseline (*p* > 0.05). After intervention, the TC had significantly lower attention bias scores than the running group and had a smaller effect (F (1, 43) = 6.023, *p* = 0.019, d = −0.42). The difference in the remaining indicators was not statistically significant (*p* > 0.05).

### 3.3. Effect of Tai Chi Exercise Intervention on the Physical Fitness of Men with MUD

As shown in Table 3, there was no significant difference in physical fitness between the TC and the running group at baseline (*p* > 0.05). After 12 weeks of Tai Chi exercise intervention, relative to the running group, a significant reduction was observed for the TC in choice reaction time (F (1, 43) = 6.181 *p* = 0.017, d = −0.72). No significant difference in diastolic blood pressure was observed (F (1, 43) = 1.378, *p* = 0.248, d = −0.41). For the blood biochemical tests, some trend to increase existed in insulin, glucose, and high-density lipoprotein cholesterol for the Tai Chi group (d = 0.2–0.5) relative to the running group. There was no significant difference in other indicators between groups (*p* > 0.05), and the effect size was less than 0.20.

## 4. Discussion

This study examined the effects of 12 weeks of Tai Chi exercise on drug cravings, attention bias, and the physical fitness of men with MUD. After 12 weeks of intervention training, Tai Chi exercise was found to be effective in improving the attention bias and reaction time among men with MUD relative to the running group. Insulin, glucose, and high-density cholesterol were increased by trend compared to the running group. In addition, men with MUD had a trend to decrease cravings compared to the running group. Therefore, this study may provide some practical references for promoting the physical and mental health in men with MUD.

### 4.1. Effects of Tai Chi on Drug Cravings in Men with MUD

Drug cravings are recognized as a central aspect of addiction, with cravings being closely associated with relapse [9]. Our study used the VAS scale to assess drug cravings [42] and found drug cravings were not significantly improved but had a trend to decrease in men with MUD after 12 weeks of Tai Chi exercise relative to the running group. While Tai Chi showed a downward trend in reducing cravings, running as an aerobic exercise also showed some effectiveness in this area. This may be due to the stress-relieving and mood-enhancing effects of running [43]. However, the mind–body integration in Tai Chi may contribute to a more significant reduction in cravings through additional mechanisms. Previous studies have shown mixed results regarding the effect of Tai Chi on drug cravings. A meta-analysis found that Tai Chi exercise was beneficial to mental health, with participants experiencing reductions in negative emotions [44]. A previous study has shown that practicing Tai Chi for three months is effective in reducing drug cravings in those using drugs [45]. Another study has shown that Tai Chi mind–body exercises reduce physiological arousal, thereby reducing cravings and relapse [46]. Furthermore, Tai Chi is a mind–body intervention exercise that includes a meditative component, and it has been shown to be beneficial in reducing cravings [45]. However, within the 12 weeks, no significant effects were observed in our study.

### 4.2. Effects of Tai Chi on Attention Bias in Men with MUD

Substance use disorders have obvious attention bias toward drugs and related stimuli [47]. Our study found that after 12 weeks of Tai Chi exercise training, attention bias scores were significantly lower in the TC than the running group. Although running is known to improve overall cognitive function [48], its effect on reducing specific attentional biases to drug-related cues was less pronounced compared to Tai Chi. The unique combination of physical exercise and mindfulness in Tai Chi may be more effective in targeting cognitive biases associated with addiction. Participants in our study could distract themselves from drug cues when faced with drug stimuli and no longer concentrate excessively on drug stimuli, which is consistent with previous studies. Tai Chi, with its gentle and smooth movements that emphasize coordination of breath and mind, is a potentially effective exercise method for improving brain health [49]. Meanwhile, previous studies have found that the practice of Tai Chi can help reduce stress and anxiety and improve concentration [50]. Four weeks of Tai Chi exercise can improve the attention bias of individuals with MUD. The mechanism might be that Tai Chi exercise can enhance attention and improve the brain’s cognitive function, thus reducing the attention bias of individuals with MUD [51]. Long-term substance abuse causes extensive harm to the brain function of individuals with MUD, leading to slow cognitive improvement [52]. However, cognitive function may improve through potential neurophysiological pathways, as Tai Chi is a multimodal mind–body exercise that requires concentration and multitasking transitions and processing [53].

### 4.3. Effects of Tai Chi on Physical Fitness in Men with MUD

A significant improvement was found in choice reaction time following the Tai Chi interventions compared with the control after 12 weeks. It was consistent with findings observed in previous research. A study in older adults with disabilities suggested that 12 weeks of wheelchair-based Tai Chi exercise significantly improved reaction times [54]. Running also showed significant positive effects on cardiovascular health and overall endurance, reinforcing its well-established role in improving physical fitness through aerobic capacity and cardiovascular conditioning [55]. However, whereas running primarily improves aerobic fitness, Tai Chi offers a more holistic approach, integrating physical, mental, and emotional elements. This integration may contribute to improvements not only in physical endurance, but also in balance, flexibility, and stress management. Therefore, Tai Chi may provide additional benefits beyond those achieved by aerobic exercise alone, particularly in populations where mental health and balance are critical concerns, such as in men with MUD. Also, in our study, a trend of decrease was found in diastolic blood pressure after 12-week interventions. Studies have shown that 12 weeks of Tai Chi exercise can significantly reduce diastolic blood pressure levels, which is consistent with the results of this study. This may be because Tai Chi exercise can improve vascular endothelial function, regulate the level of hormones in the blood, and maintain the relative stability of the internal environment, which makes the pressure in the blood vessels balanced to a lower blood pressure [56].

However, several indicators did not show significant changes after the intervention. Systolic blood pressure showed only a small reduction, possibly because the 12-week period was too short to have a significant effect on blood pressure. Also, systolic pressure may be more resistant than diastolic pressure, requiring longer interventions to see significant effects [57]. Lung capacity remained largely unchanged, probably because significant improvements in lung capacity are difficult to achieve in the short term. Improving lung function may require a longer intervention period to see significant changes [58,59]. Grip strength also remained largely unchanged, which may be due to Tai Chi’s focus on balance, flexibility, and breathing coordination rather than direct strength training [60]. One-leg standing with eyes closed showed a non-significant trend towards improvement, suggesting that improvements in balance may require longer-term practice, as initial balance ability had a significant impact on intervention outcomes [61]. Studies have shown that initial balance ability has a significant impact on balance training outcomes, and individuals with lower baseline balance ability may require longer or more targeted interventions to see significant improvements [62]. Triglyceride and low-density lipoprotein cholesterol (LDL-C) levels were minimally affected, suggesting that the specific physiological adaptations required to significantly affect these lipid profiles may take longer or require a more intense exercise program. In addition, individual variability in lipid metabolism may also play a role [63]. Total cholesterol levels also did not change significantly, suggesting that longer durations may be required to see meaningful improvements in lipid profiles [64].

Despite the lack of significant change in some indicators, others showed positive trends. Chronic drug abuse can lead to individuals with substance use disorder being more likely to be at high risk of developing metabolic syndrome [65]. Studies have shown that Tai Chi practice improves cardiovascular health, regulates blood sugar and lipid levels, and enables the body to better metabolize glucose and lipids [66,67]. In this study, the TC had a certain improvement trend in insulin, glucose, and high-density cholesterol compared with the running group after 12 weeks of Tai Chi intervention. A meta-analysis showed long-term Tai Chi exercise regulates blood sugar in diabetics [68]. Drug abuse can cause severe damage to cardiovascular function, while high-density cholesterol can protect blood vessels by binding to receptors in the vascular endothelium to stimulate the release of eNOS [69]. The present study found that after 12 weeks of Tai Chi exercise intervention, the high-density cholesterol of men with MUD showed an increased trend. A study has demonstrated that practicing Tai Chi for three months can enhance levels of high-density lipoprotein in individuals with cardiovascular disease [70]. Therefore, a 12-week Tai Chi exercise intervention may help improve the ability of glucolipid metabolism regulation, improving cardiovascular function.

Some increase was found in waist circumference and BMI following the 12-week Tai Chi training interventions compared with the running group. Neurobiological and imaging research suggested that the mechanism may be the shared pathways of food and drug intake. There has been a hypothesis that food and drugs compete for the same brain reward sites. A negative correlation between BMI and drug use has been proved [71]. In our study, the men with MUD had been clean for a long period and therefore showed an increasing trend in BMI and waist circumference.

## 5. Conclusions

The 12-week Tai Chi intervention improved attention bias and reaction time in men with MUD compared to the running group. Drug cravings, diastolic blood pressure, and glucolipid metabolism function had a trend to change. Tai Chi exercise might be a potential auxiliary method for the rehabilitation of individuals with MUD. Clinically, Tai Chi could be incorporated into rehabilitation programs to enhance physical and psychological recovery. Practically, it offers a low-cost, low-risk, and accessible form of exercise for a variety of settings. Although significant positive effects were found, more studies are needed to further explore the underlying mechanisms of the effect of Tai Chi on physical and psychological recovery in this population.

## 6. Strengths, Limitations, and Future Directions

There were several strengths to this study. First, Tai Chi is an excellent traditional Chinese exercise that can promote physical and mental recovery for individuals struggling with addiction. Tai Chi has been shown to positively affect drug cravings, attention bias, and physical health in men with MUD. Second, the intervention was based on Tai Chi exercise and lasted 12 weeks. Training intensity was monitored throughout the course to ensure the quality and safety of training. Third, in this study, an active aerobic exercise control was set up to perform treadmill exercise. This was done to better explore the effects of Tai Chi exercise intervention on the physical and mental health in men with MUD.

However, several limitations of our study should be considered. First, although Tai Chi has shown promise in reducing drug cravings, our study did not observe significant effects within the 12-week intervention period. This suggests that a longer intervention period may be required to fully understand the potential effects of Tai Chi on drug cravings in this population. In addition, this study did not include a follow-up period, which limits our ability to assess the long-term effects of the exercise interventions. Future studies should extend the follow-up period to better test the long-term effects of such interventions. Second, only men with MUD were selected for this study, which limits the generalizability of the findings. Future research should include women with MUD to allow comparative analyses between genders.

Previous studies have shown mixed results regarding the effect of Tai Chi on drug cravings, suggesting the need for further research. Neuropsychologically, Tai Chi is thought to affect brain regions associated with emotion regulation and stress response, such as the prefrontal cortex and the amygdala. Mind–body interventions such as Tai Chi are thought to enhance neuroplasticity and improve connectivity within these brain regions, potentially leading to reduced cravings over time. To better understand these neuropsychological mechanisms, future research should focus on longer intervention periods and more rigorous adherence monitoring. Additionally, broadening the assessment to include a wider range of psychological, social, and emotional factors would provide a broader understanding of Tai Chi’s effects. Advanced neuroimaging techniques such as functional near-infrared spectroscopy (fNIRS) and electroencephalography (EEG) could also be used to explore the underlying mechanisms of the observed effects.

## Figures and Tables

**Table 1:** Basic information of participants (M (SD)/n (%)).

Variable	TC	RG	*t*/χ^2^	*p*
n	20	24		
Age (M (SD))	38.30 (9.07)	39.70 (8.56)	0.560	0.578
Years of education (M (SD))	9.05 (2.11)	8.83 (3.90)	0.234	0.816
Marital status (n, %)			3.020	0.221
Single	5 (25.00)	8 (33.33)		
Married	10 (50.00)	6 (25.00)		
Divorced	5 (25.00)	10 (41.67)		
Years of methamphetamine use (M (SD))	8.63 (5.35)	9.00 (6.17)	0.208	0.836
MMSE score (M (SD))	28.80 (1.64)	27.71 (2.74)	1.561	0.126

Note: TC, Tai Chi group; RG, running group; MMSE: the Mini-Mental State Examination; M (SD): mean (standard deviation).

**Table 2:** Comparison of attention bias results between Tai Chi and running groups (M (SD)).

Variable		TC (n = 20)		RG (n = 24)		TC vs. RG			
		0 Weeks	12 Weeks	0 Weeks	12 Weeks	F (1, 43)	*p*	d	ES
Visual dot-probe paradigm	Attention bias score	4.08 (31.78)	−3.56 (23.10)	5.94 (33.07)	13.82 (22.29)	6.023	0.019 *	−0.42	S
	Reaction time of neutral picture, ms	440.00 (66.09)	448.36 (61.43)	422.02 (54.76)	448.89 (56.02)	1.474	0.232	−0.43	S
	Reaction time of drug picture, ms	435.92 (66.33)	451.92 (65.73)	416.08 (54.95)	435.07 (51.48)	0.020	0.887	−0.07	-

Note: * *p* < 0.05; TC, Tai Chi group; RG, running group; ES, effect size: none (0 ≤ d < 0.20), small effect (0.20 ≤ d < 0.50), medium effect (0.50 ≤ d < 0.80), large effect (d ≥ 0.80); M (SD): mean (standard deviation).

**Table 3:** Comparison of physical fitness results between Tai Chi and running groups (M (SD)).

Variable	TC		RG		TC vs. RG			
	0 Weeks	12 Weeks	0 Weeks	12 Weeks	F (1, 43)	*p*	d	ES
Waist circumference	85.70 (8.16)	90.95 (8.23)	84.79 (7.03)	86.08 (6.00)	7.433	0.010 *	0.68	M
BMI, kg/m^2^	24.06 (3.05)	24.96 (3.23)	23.42 (2.90)	24.07 (3.49)	0.580	0.451	0.22	S
Systolic pressure, mmHg	125.75 (15.40)	123.60 (12.96)	123.04 (18.37)	120.29 (16.00)	0.338	0.564	0.04	-
Diastolic pressure, mmHg	73.85 (10.40)	68.75 (9.89)	72.13 (13.14)	70.75 (12.72)	1.378	0.248	−0.41	S
Lung capacity, mL	2791.38 (1041.31)	2797.25 (858.47)	2323.57 (540.02)	2342.43 (676.63)	0.449	0.508	−0.03	-
Grip strength, kg	42.54 (7.20)	42.61 (6.30)	40.18 (6.70)	40.36 (5.87)	0.075	0.786	−0.03	-
Choice reaction time, s	0.54 (0.09)	0.52 (0.08)	0.54 (0.10)	0.57 (0.09)	6.181	0.017 *	−0.72	M
One-leg standing with eyes closed, s	8.98 (8.40)	10.41 (5.43)	9.70 (8.17)	12.23 (7.50)	0.387	0.539	−0.14	-
Insulin, mmol/L	8.85 (3.89)	8.55 (3.54)	6.64 (3.74)	8.28 (5.10)	0.519	0.476	−0.46	S
Glucose, mmol/L	3.63 (0.56)	4.32 (0.43)	3.28 (0.46)	4.45 (0.45)	0.721	0.401	−0.74	M
Total cholesterol, mmol/L	3.83 (1.06)	3.89 (1.08)	3.48 (1.00)	3.70 (1.06)	0.595	0.445	−0.11	-
Triglyceride, mmol/L	1.23 (0.65)	1.31 (0.62)	0.88 (0.39)	0.95 (0.49)	4.025	0.052	0.02	-
HDL-C, mmol/L	0.66 (0.53)	1.10 (0.19)	0.92 (0.47)	1.14 (0.20)	0.601	0.443	0.40	S
LDL-C, mmol/L	2.26 (0.92)	2.35 (0.89)	1.94 (0.79)	2.19 (0.87)	0.648	0.426	−0.13	-

Note: * *p* < 0.05; TC, Tai Chi group; RG, running group; ES, effect size: none (0 ≤ d < 0.20), small effect (0.20 ≤ d < 0.50), medium effect (0.50 ≤ d < 0.80), large effect (d ≥ 0.80); M (SD): mean (standard deviation); HDL-C: high-density cholesterol; LDL-C: low-density cholesterol.

## Data Availability

The data presented in this study are available on request from the corresponding author.
